# Ferrimagnetic Vortex Nanorings Facilitate Efficient and Safe Deep‐Brain Magnetothermal Stimulation in Freely Moving Mice

**DOI:** 10.1002/EXP.20240118

**Published:** 2025-12-04

**Authors:** Galong Li, Xin Qiao, Yu Zhao, Dongyan Li, Guigen Zhang, Xiaoli Liu, Fulin Chen, Huaning Wang, Hongbing Lu, Jin Zhou, Changyong Wang, Haiming Fan

**Affiliations:** ^1^ College of Chemistry and Materials Science Key Laboratory of Synthetic and Natural Functional Molecule of Ministry of Education Northwest University Xi'an China; ^2^ School of Biomedical Engineering Shaanxi Provincial Key Laboratory of Bioelectromagnetic Detection and Intelligent Perception Air Force Medical University Xi'an China; ^3^ Beijing Institute of Basic Medical Sciences Beijing P. R. China; ^4^ Department of Biomedical Engineering University of Kentucky Lexington Kentucky USA; ^5^ Department of Psychiatry Xijing Hospital Air Force Medical University Xi'an China

**Keywords:** magnetic nanomaterials, magnetic neural stimulation, magnetogenetics, magnetothermal effect, wireless neuromodulation

## Abstract

Magnetothermal neuromodulation is a minimally invasive, deep‐brain accessible, and tether‐free technique. The precisely timed activation of thermosensitive ion channels, such as TRPV1, with local heat generated using magnetic nanoparticles is crucial for efficient neuromodulation. Nevertheless, this technique is greatly hindered by its long stimulus‐response time and high safety risks due to the poor heat‐generating performance of the nanomediators. Herein, we report the establishment of a ferrimagnetic vortex iron oxide nanoring (FVIO)‐mediated magnetothermal neurostimulation technique that is efficient and safe. Compared with widely used superparamagnetic iron oxide nanomediators (SPIOs), the FVIOs triggered Ca^2+^ influx into HEK293T cells and cortical neurons at an Fe concentration of 51 µg mL^−1^, which is 20.27‐fold lower than that needed for SPIOs. In vivo magnetothermal stimulation in the central nucleus of the amygdala of mice further demonstrated that FVIOs with the optimal dose of 0.05 µg evoked fear behaviors with an average latency of 2.51 s, which was 2.3‐fold faster than that in the SPIO (0.80 µg)‐treated group. More importantly, FVIOs‐mediated stimulation not only exhibited negligible histopathological alterations and proinflammatory cytokine expression but also successfully elicited fear behaviors in transgene‐free mice. The FVIO‐mediated efficient and safe neuromodulation has the potential for future neuroscience exploitation and neurological disease treatment.

## Graphical abstract

Magnetothermal neuromodulation is greatly hindered by its long stimulus‐response time and high safety risks due to the poor heat‐generating performance of the nanomediators. We report the establishment of a novel ferrimagnetic vortex iron oxide nanoring (FVIO)‐mediated efficient and safe magnetothermal neurostimulation. The FVIOs at remarkably low dosage triggered Ca^2+^ influx into cells and quickly evoked fear behaviors in freely moving mice.

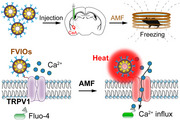

## 1 Introduction

Magnetothermal neurostimulation is emerging as a powerful technique for gaining insight into intricate brain circuits and performing therapeutic investigations of neurological disorders, given its distinct advantages, such as minimal invasiveness, tether‐free operation, deep brain accessibility, and high spatial resolution [1, 2]. In this technique, biocompatible magnetic nanoparticles are utilized as nanoheaters to generate heat locally through the hysteresis loss process under an alternating magnetic field (AMF) [3]. The increase in local temperature can activate the thermosensitive ion channels that are overexpressed on the cell membrane and trigger an influx of calcium (Ca^2+^) into neurons, thereby modulating neural activity [4]. To date, magnetothermal neurostimulation has been demonstrated to bidirectionally modulate neuronal activity [5, 6, 7, 8], successfully regulate blood glucose [9, 10] and adrenal hormone levels in vivo [11], and control the motor behaviors of worms [8, 12], flies, [13] and freely moving mice [14]. However, current techniques still suffer from low activation efficiency and high required dosages of nanoheaters, leading to an unexpectedly long stimulus‐response time and potential safety concerns [13].

According to the principles of magnetothermal neurostimulation, the precisely timed activation of a single neuron depends on both the expression level of thermosensitive ion channels and the heating efficiency of the nanoparticles under an AMF [11, 15, 16]. Transient receptor potential cation channel subfamily V member 1 (TRPV1), the thermosensitive ion channel most commonly used in neuromodulation, can be activated by local heat (>43°C)‐induced conformational changes in the channel pore [17, 18, 19, 20]. Moreover, the rate of TRPV1 opening increases exponentially with increasing surrounding temperature [19, 21]. Because an increase in TRPV1 expression in vivo requires viral vector‐mediated gene transfection, which comes with high safety risks, the key to quickly and safely activating TRPV1 lies in boosting the heat‐generating performance of magnetic nanoparticles [3, 4, 11]. Unfortunately, the superparamagnetic iron oxides (SPIOs) and ferritin protein widely used in magnetothermal neurostimulation show poor thermal conversion efficiency due to their superparamagnetic nature and minimal hysteresis loss [22, 23, 24, 25]. In addition, an AMF with a high amplitude (*H*) and frequency (*f*) is frequently needed to improve the heat‐generating ability of the nanoheater [26, 27, 28]. Nevertheless, less improvement has been achieved because of the biologically acceptable limit (H × *f* ≤ 5 × 10^9^ A (m s)^−1^) [28, 29, 30]. Recognizing the above limitations, developing a high‐performance magnetic nanoparticle‐mediated stimulation technique is a valuable approach for realizing efficient and safe magnetothermal neuromodulation [31, 32].

Significant efforts have been made to synthesize various magnetic nanoparticles with enhanced heat‐generating performance given their wide application in magnetic hyperthermia, controlled drug delivery, cell signal transduction activation, and magnetothermal neurostimulation [30, 33, 34, 35, 36, 37, 38]. The thermal conversion efficiency of nanoparticles is commonly evaluated using the specific absorption rate (SAR), which mainly depends on the intrinsic magnetic properties of the nanoparticles [39, 40, 41]. Despite tuning their composition, shape, size, and surface chemistry of various magnetic nanoparticles, most exhibit relatively low SAR values ranging from 250–1000 W g^−1^ [30, 42, 43]. As a result, these nanoparticles take more time to reach the threshold temperature and activate TRPV1, leading to long latency times (≈14.7 s) for behavior onset [13, 18, 44]. Applying a high dose of magnetic nanoparticles is an alternative method to reduce the heating time [26]. Nevertheless, using excessive amounts of nanoheaters increases the risk of non‐negligible safety issues in the brain, such as dopamine neuron damage, homeostatic disruption, and inflammatory responses [45]. We previously reported that novel biocompatible ferromagnetic vortex‐domain iron oxide nanorings (FVIOs) exhibit an ultrahigh SAR of greater than 3000 W g^−1^ because of their unique vortex‐to‐onion magnetization reversal phenomenon upon AMF exposure [46]. Notably, as our simulation results revealed, only 8.70 × 10^−6^ ng of FVIOs could effectively increase the local temperature from 37°C to 43°C, whereas 33.6 × 10^−6^ ng of SPIOs was needed under the same AMF conditions (Figure S1). In addition, relatively large size and biocompatible FVIOs are more stable in the brain, which is favorable for long‐term and repeated magnetothermal stimulation in vivo [46]. As such, FVIOs are likely to be high‐performance nanoheaters that facilitate quick activation of TRPV1, which might provide an opportunity to establish a highly efficient and safe magnetothermal neurostimulation technique.

In the present study, we comprehensively investigated FVIO‐facilitated magnetothermal neurostimulation in vitro and in vivo, and compared the results with those of widely used SPIO‐based approaches. The newly designed FVIOs with an anti‐His antibody coating triggered Ca^2+^ influx in TRPV1‐expressing HEK293T cells and cortical neurons with a minimum Fe concentration of 51 µg mL^−1^, which was 20.27‐fold lower than that of the SPIOs under the same AMF treatment. In vivo magnetothermal stimulation in the central nucleus of the amygdala (CeA) further demonstrated that FVIO treatment quickly evoked fear behaviors in mice, exhibiting a response time that was 2.3‐fold faster than that in the SPIO‐treated group. Overall, the lowest effective dose of FVIOs was 0.05 µg in vivo, 16.7‐fold less than the effective dose of SPIOs. More importantly, the SPIO‐treated mice exhibited significant histopathological changes (such as vacuolar degeneration) and upregulated proinflammatory cytokine (such as interleukin‐6 (IL‐6)) expression in the CeA. In contrast, the FVIO‐treated mice showed negligible histopathological alterations and changes in proinflammatory cytokine expression. In addition, in the FVIO‐treated groups, fear behaviors were successfully induced in the mice on the 60^th^ day after injection. However, in the SPIO‐treated group, fear behaviors could not be induced on the 30^th^ day. Notably, only 0.28 µg of FVIOs activated the CeA endogenously expressing TRPV1 and elicited fear behaviors in transgene‐free mice. The aim of this work is to establish an efficient and safe FVIO‐facilitated magnetothermal neuromodulation technique, which is expected to be a powerful tool for future neuroscience investigations and therapeutic applications to neurological disorders.

## 2 Results and Discussion

### 2.1 Synthesis and Characterization of FVIOs

**FIGURE 1:**
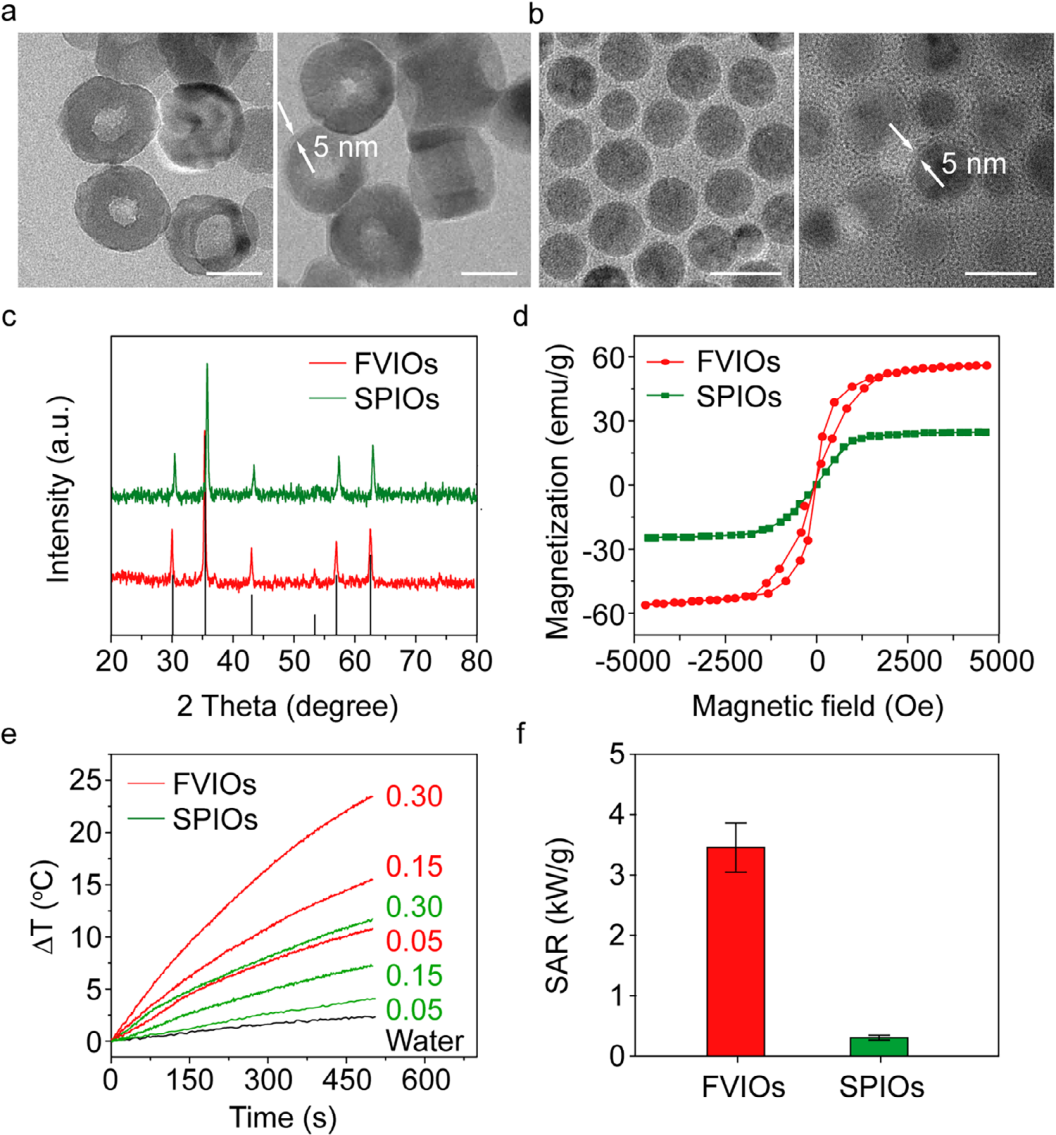
Characterization of FVIOs and SPIOs. (a) TEM images of the oleic acid‐coated FVIOs (left) and the negatively stained FVIOs (right). (b) TEM images of the oleic acid‐coated SPIOs (left) and the negatively stained SPIOs (right). The marked surface layer (≈5 nm thick) corresponds to the anti‐His antibodies and DHCA coatings. Scale bars, 20 nm. (c) Powder X‐ray diffraction patterns of the SPIOs and FVIOs. (d) Room temperature magnetization curves constructed for SPIOs and FVIOs. (e) Temperature changes in the SPIOs and FVIOs solutions at various Fe concentrations (0.05, 0.15, and 0.30 mg mL^−1^) under AMF exposure (*H* = 20 mT and *f* = 275 kHz). (f) The specific absorption rate (SAR) was calculated from the rate of temperature increase of the SPIO and FVIO solutions. The data are presented as the mean ± standard deviation; *n* = 3 independent experiments for each sample.

The 3,4‐dihydroxyhydrocinnamic acid (DHCA)‐coated FVIOs (DHCA‐FVIOs) with an average diameter of 52 nm were first synthesized using a wet chemical method reported previously [46]. Moreover, 20 nm DHCA‐SPIOs were prepared as controls in this study [47]. Scanning electron microscopy (SEM) revealed that the DHCA‐FVIOs had a uniform ring shape with an average outer diameter, inner diameter, and thickness of 52, 18, and 15 nm, respectively (Figure S2a). For precisely targeted magnetothermal stimulation, DHCA‐FVIOs and DHCA‐SPIOs were further covalently bound to anti‐His antibodies using a 1‐ethyl‐3‐(3‐dimethylaminopropyl)carbodiimide/*N*‐hydroxysuccinimide (EDC/NHS) coupling reaction to enable TRPV1 targeting via an extracellular 6× His epitope tag on the transfected cells. The transmission electron microscopy (TEM) images of the negatively stained FVIOs and SPIOs modified with anti‐His antibodies are displayed in Figure 1a,b. The antibody coatings are observed as a marked layer (≈5 nm thick) on the surface of the nanoparticles. The covalent binding of the antibodies to the nanoparticles was confirmed using infrared spectroscopy and zeta potential measurement (Figure S2b,c). Moreover, the powder X‐ray diffraction (XRD) patterns (Figure 1c) confirmed the cubic inverse spinel phase of Fe_3_O_4_ in both FVIOs and SPIOs. As shown in Figure S3a, after anti‐His antibody conjugation, the anti‐His‐FVIOs and anti‐His‐SPIOs show hydrodynamic diameters of 105.71 and 37.84 nm, respectively. In addition, both samples maintained their original hydrodynamic diameters after incubation in phosphate buffered saline (PBS) for more than 80 days, suggesting their good colloidal stability (Figure S3b). The magnetic properties of these two samples were also examined using a vibrating sample magnetometer (VSM). Compared with SPIOs, FVIOs exhibited a vortex magnetic structure with a much greater saturation magnetization and larger hysteresis loop area at room temperature (Figure 1d). Induction heating measurements were then performed with the FVIOs using an induction heating system (an AMF setup, Supermag M5), in which the temperature changes in the samples were recorded by an optical fiber probe. And the applied AMF had a frequency (*f*) of 275 kHz and an amplitude (*H*) of 20 mT. Figure 1e presents the temperature profiles of the anti‐His‐FVIOs and anti‐His‐SPIOs aqueous suspensions with Fe concentrations of 0.05, 0.15, and 0.30 mg mL^−1^. The calculated SAR of the FVIOs was approximately 3462 W g^−1^, which is 10.78 times greater than that of the SPIOs (321 W g^−1^) (Figure 1f). The superior heat‐generating ability of the FVIOs can be attributed to the significantly increased hysteresis loss during the vortex‐to‐onion magnetization reversal process in response to AMF exposure [48].

Cytotoxicity evaluations were further performed with anti‐His‐FVIOs and anti‐His‐SPIOs with Fe concentrations ranging from 0 to 1000 µg mL^−1^ using HEK293T cells. After 24 h of incubation, the FVIOs and SPIOs did not alter the viability of HEK293T cells or cortical neurons at Fe concentrations ranging from 0 to 200 µg mL^−1^ (Figure S4a,b). When the Fe concentration increased from 200 to 1000 µg mL^−1^, both the FVIOs and SPIOs exhibited severe cytotoxicity with significant decreases in cell viability (much lower than 80% viability). These results imply that the biocompatible anti‐His‐FVIOs with superior heat induction properties can be successfully prepared as efficient magnetothermal transducers for neurostimulation applications.

### 2.2 In Vitro Magnetothermal Activation of TRPV1‐Expressing Cells

**FIGURE 2:**
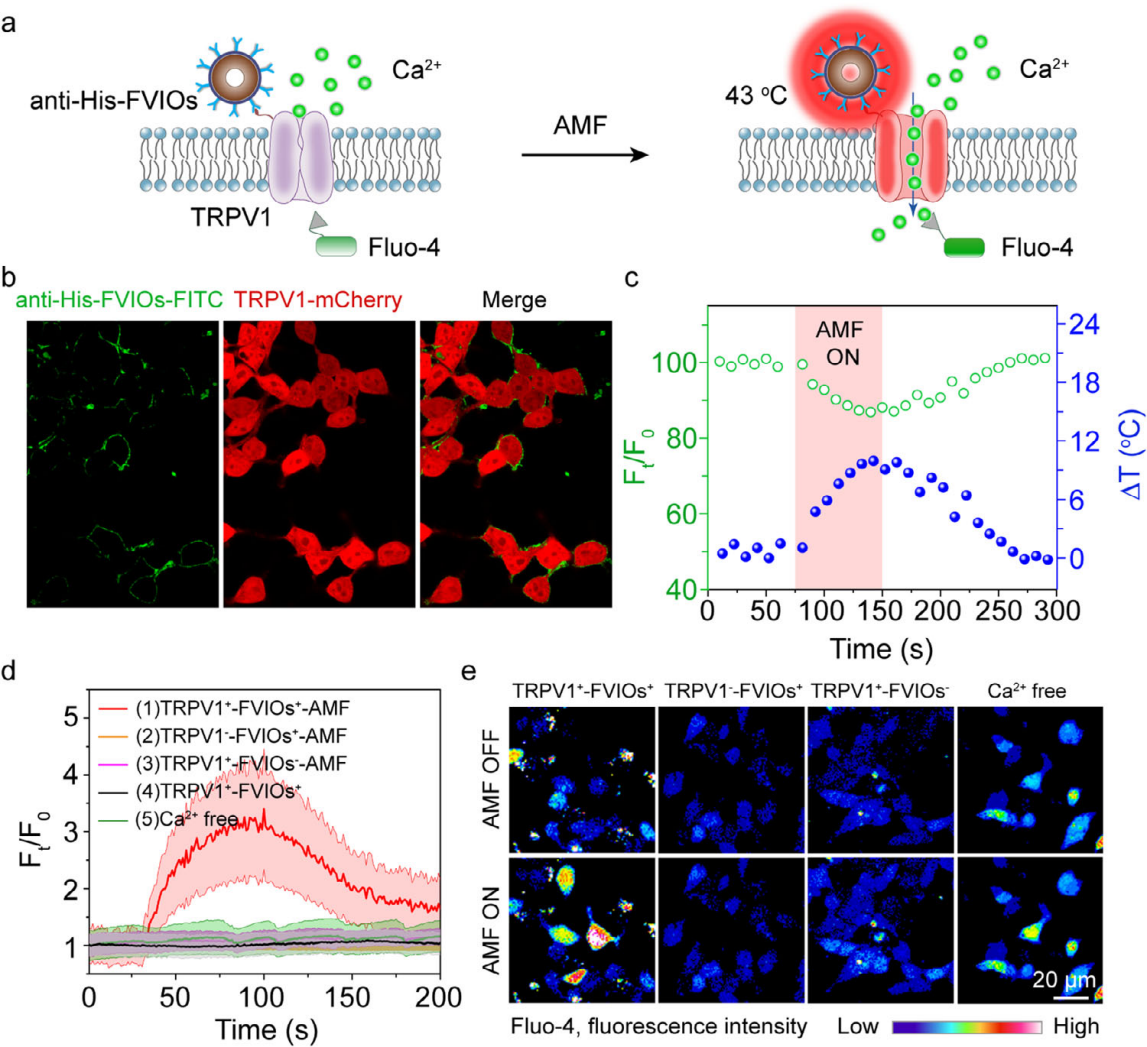
In vitro magnetothermal activation of TRPV1 triggers Ca^2+^ influx. (a) Schematic of FVIO‐mediated magnetothermal activation of TRPV1 triggering of Ca^2+^ influx. Fluo‐4 was used as the Ca^2+^ indicator. (b) Structured illumination microscopy (SIM) images of TRPV1‐expressing HEK293T cells (red) bound to FL labeled anti‐His‐FVIOs (green). (c) Local temperature increases near the FVIOs upon AMF exposure determined by measuring the change in green fluorescence intensity of anti‐His‐FVIOs‐ FL target the cell membrane. (d) Fold changes in fluorescence intensity (*F_t_*/*F*_0_ values) and (e) color maps of Fluo‐4 in HEK293T cells under different experimental conditions. TRPV1‐expressing, TRPV1^+^; TRPV1‐nonexpressing, TRPV1^−^; before AMF exposure, AMF OFF; during AMF exposure, AMF ON; with FVIOs treatment, FVIOs^+^; without FVIOs treatment, FVIOs^−^.; Ca^2+^ free group, TRPV1^+^‐FVIOs^+^‐AMF‐Ca^2+^‐free group. AMF conditions: H = 20 mT and *f* = 290 kHz. The solid lines and shaded areas represent the means and standard error of the mean (s.e.m), respectively, the data presented as the mean ± s.e.m.

To precisely evaluate the efficacy of FVIO‐mediated magnetothermal activation of the TRPV1 channel, monoclonal HEK293T cell lines were generated that stably expressed 6 × His‐labeled TRPV1 channels. TRPV1 was labeled with the red fluorescent protein mCherry, which was separated by the posttranscriptional cleavage linker p2A. The western blotting showed that TRPV1 fusion protein was expressed on HEK293T cells (Figure S5). Figure 2a presents a schematic of the FVIO‐mediated magnetothermal activation of TRPV1 and Ca^2+^ influx into cells. The fluorescent Ca^2+^ indicator Fluo‐4 was loaded into transfected HEK293T cells to monitor the changes in the intracellular Ca^2+^ concentration in response to magnetothermal stimulation. After application of the anti‐His‐FVIOs targeting the TRPV1 channel, the change in green fluorescence intensity during magnetothermal stimulation was recorded in real time using confocal laser scanning microscopy (CLSM) equipped with an AMF generator (Figure S6).

Before performing the magnetothermal activation experiments, we measured the local change in temperature around the FVIOs under an AMF (20 mT, 200 kHz) to confirm that the local heating offered by the FVIOs was sufficient to activate the TRPV1 channel. FVIOs were modified with fluoresceinamine (FL; emits green fluorescence) for use as a molecular‐scale thermometer. First, the fluorescence intensity and bulk temperature increase of the FVIOs‐FL dispersion were recorded during AMF exposure. The percent change in fluorescence intensity decreased linearly with increasing temperature [8], reaching –1.97 ± 0.04%/°C for FL (Figure S7). On this basis, the FVIOs‐FL modified with antibodies were used to target the TRPV1 channels expressed on cultured HEK293T cells (Figure 2b; Figure S8), and the change in FL fluorescence intensity was measured upon AMF exposure. The local temperature changes near the FVIOs were calculated according to the linear temperature‐fluorescence intensity curve. After 30 s of AMF exposure, the local temperature in the immediate vicinity of TRPV1 on the cell membrane increased from 36°C to 43°C and then decreased rapidly to 36°C in the absence of an AMF (Figure 2c). A local temperature increase (more than 43°C) near the FVIOs is sufficient to activate the TRPV1 channel by inducing a conformational change in the protein. In addition, the simulation results revealed that the local temperature decreases as the distance increases between the surface of a single FVIO and water (Figure S9a). No ambient heating effect was generated in the cell culture media according to the infrared thermal images (Figure S9b).

FVIO‐mediated magnetothermal activation of TRPV1 was systematically investigated through in situ Ca^2+^ imaging in cultured 293T cells, where Ca^2+^ influx indicates the opening of the TRPV1 channel and increased cellular activity. The fold changes in the intracellular fluorescence intensity (*F_t_*/*F*_0_) of the Ca^2+^ indicator Fluo‐4 were recorded under different experimental conditions. Intense green fluorescence corresponding to the intracellular Ca^2+^ signal was observed upon AMF exposure. After treatment with anti‐His‐FVIOs, the *F_t_*/*F*_0_ of the TRPV1‐expressing HEK293T cells increased with prolonged AMF exposure, which was attributed to successful heat‐triggered Ca^2+^ influx, as shown in Figure 2d,e. This increase in fluorescence induced by Ca^2+^ influx was observed only in TRPV1‐expressing cells that were activated by anti‐His‐FVIOs‐mediated magnetothermal stimulation and in the group treated with agonist capsaicin (CAP, 10 µM). The cells in the negative control group were cultured in media containing the antagonist capsazepine (CZP, 5 µM), and no significant difference in fluorescence intensity was observed during anti‐His‐FVIO‐mediated magnetothermal stimulation. Moreover, the control groups, including the TRPV1 non‐expressing group, the FVIO‐treated group, and the AMF‐treated group, exhibited negligible Ca^2+^‐dependent changes in fluorescence intensity.

**FIGURE 3:**
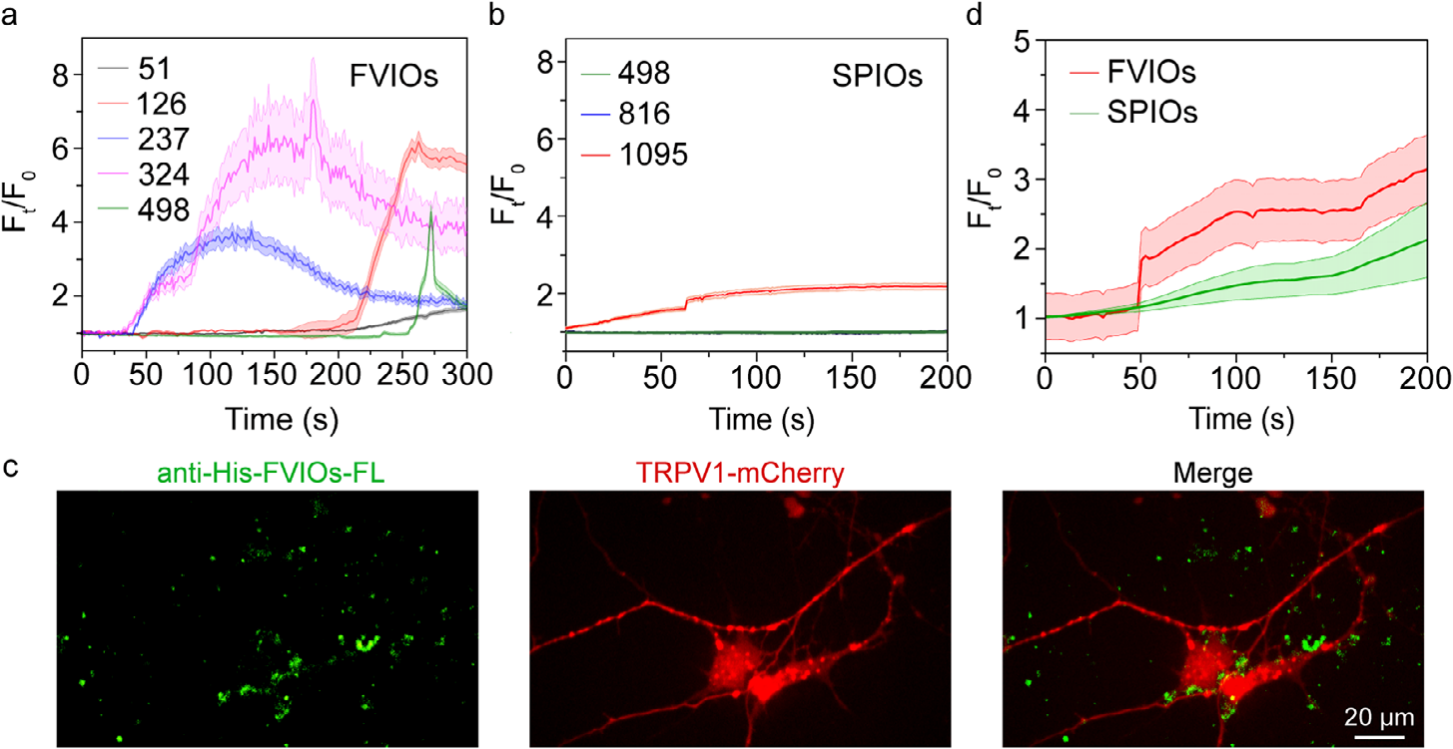
In vitro magnetothermal induction of Ca^2+^ influx in TRPV1‐expressing 293T cells. (a,b) Fold changes in fluorescence intensity (*F_t_*/*F*_0_ values) of Fluo‐4 in 293T cells during magnetothermal treatment with anti‐His‐FVIOs at Fe concentrations ranging from 51 to 498 µg mL^−1^, and with anti‐His‐SPIOs at Fe concentrations of 498, 816, and 1095 µg mL^−1^. AMF conditions: H = 20 mT and *f* = 290 kHz. (c) SIM images of TRPV1‐expressing cortical neurons (red) bound to anti‐His‐FVIO‐FL (green). (d) Fold changes in fluorescence intensity (*F_t_*/*F*_0_ values) of Fluo‐4 as a function of time for TRPV1‐expressing cortical neurons treated with FVIOs (324 µg mL^−1^) or SPIOs (1095 µg mL^−1^). AMF conditions: H = 20 mT and *f* = 290 kHz. The data are presented as the mean ± s.e.m.; the solid lines indicate the means, and the shaded areas indicate the s.e.m.

We further studied the influence of the Fe concentration on the stimulus‐response time and fold changes in fluorescence intensity due to Ca^2+^ influx into cells. As shown in Figure 3a, the response time of Ca^2+^ influx into cells decreased when the Fe concentration from the anti‐His‐FVIOs in the HEK293T cell culture media ranged from 51 to 498 µg mL^−1^. HEK 293T cells treated with anti‐His‐FVIOs at an Fe concentration of 324 µg mL^−1^ showed the fastest response in terms of Ca^2+^ influx and the greatest fold change in fluorescence intensity (Movie S1). Ca^2+^ influx into cells occurred only at an Fe concentration of 1095 µg mL^−1^ or greater in the SPIO‐treated groups (Figure 3b). Notably, the minimum Fe concentration needed for TRPV1 activation was 51 µg mL^−1^ in the FVIO‐treated group, which was 20.27‐fold lower than that in the SPIO‐treated group. This finding is consistence with our early simulation results showing that a single TRPV1 channel can be activated using a less FVIOs than SPIOs (Figure S1). Together, this experimental evidence suggests that FVIOs with superior heat‐generating performance could enable efficient magnetothermal activation of thermosensitive TRPV1 expressed on cells.

We further examined Ca^2+^ influx in cultured TRPV1‐expressing cortical neurons using the nanoheaters anti‐His‐FVIOs. FL‐labeled anti‐His‐FVIOs successfully targeted TRPV1 on cortical neurons (Figure 3c). Moreover, the cells were loaded with Fluo‐4 to monitor Ca^2+^ influx (Figure S10). As shown in Figure 3d, upon magnetothermal stimulation with anti‐His‐FVIOs at an Fe concentration of 324 µg mL^−1^, Ca^2+^ fluorescence in cortical neurons rapidly increased from 50 to 200 s (Movie S2). In contrast, the Ca^2+^ fluorescence intensity in neurons increased slowly in response to magnetothermal stimulation by the anti‐His‐SPIOs at an Fe concentration of 1095 µg mL^−1^. Therefore, the anti‐His‐FVIO nanoheater is believed to enable efficient magnetothermal neural activation at low Fe concentrations.

### 2.3 FVIOs‐Mediated Fast and Safe Magnetothermal Neurostimulation In Vivo

**FIGURE 4:**
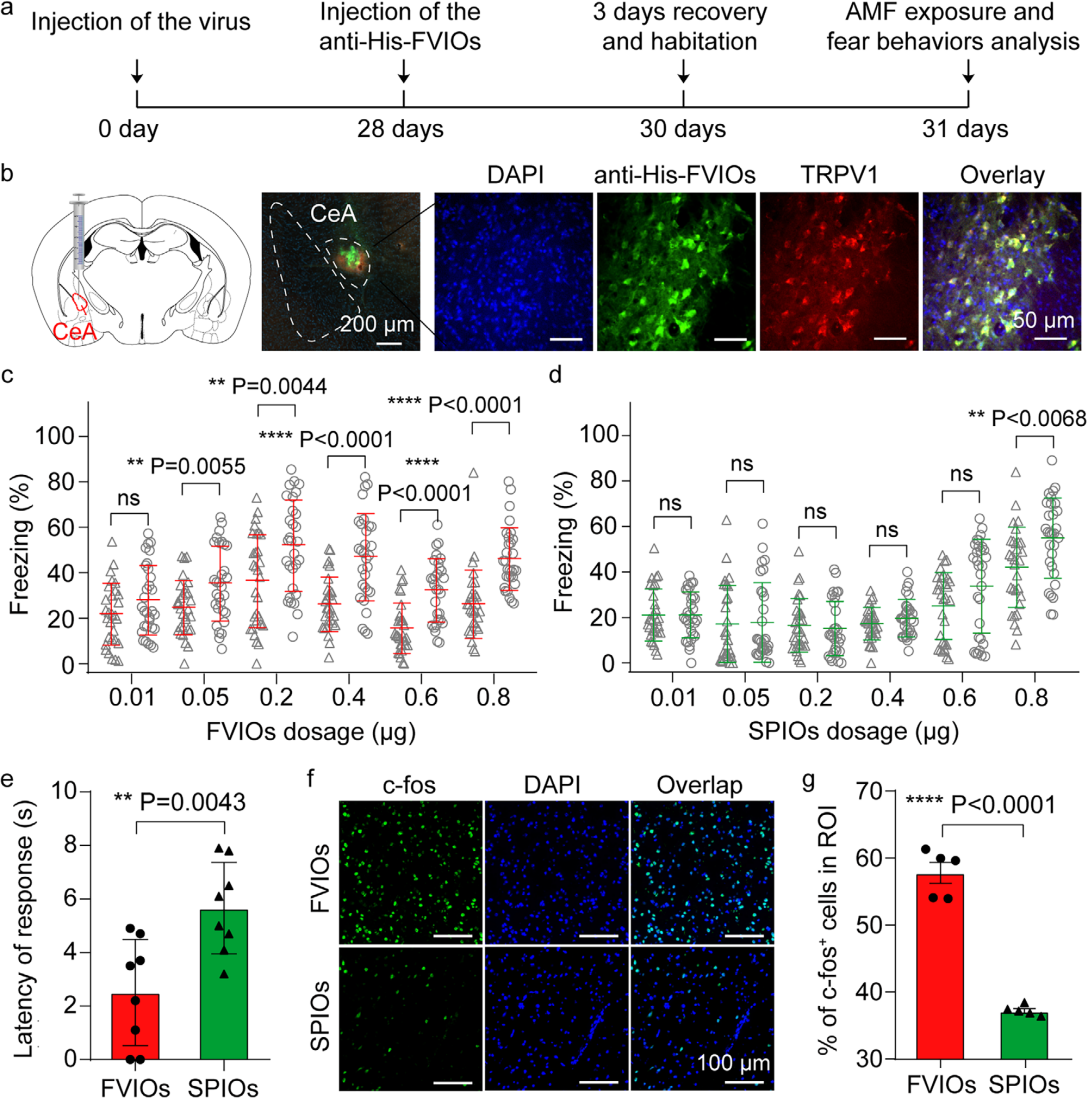
Virus and FVIOs injection into the mouse CeA for the magnetothermal neuromodulation of fear behaviors. (a) Scheme of anti‐His‐FVIO‐based magnetothermal neurostimulation of the CeA and fear behavior analysis of freely moving mice. (b) Confocal images of brain slices showing that the anti‐His‐FVIOs‐FL (green) are located in the TRPV1‐expressing CeA regions (red) of the mice. (c,d) Percentage of freezing time in mice after injection with different doses of anti‐His‐FVIOs (or anti‐His‐SPIOs) before and during AMF stimulation (H = 20 mT, *f* = 275 kHz). The black and red (or green) lines represent AMF OFF and AMF ON, respectively. One‐way ANOVA was performed, followed by a two‐sided Student's test with thresholds. (e) Latency to the start of the first freezing episode during AMF stimulation in mice. (f) DAPI (blue), c‐fos (green), and overlay confocal images of the CeA regions of mice obtained after magnetothermal stimulation. (g) Percentage of c‐fos‐expressing cells in the CeA among the DAPI‐labeled cells. Error bars indicate the mean ± s.e.m. (*n* = 5), two‐sided Student's *t*‐test with the threshold ****p < 0.0001.

Next, we verified that anti‐His‐FVIO‐mediated magnetothermal stimulation in vivo can efficiently and safely evoke quick behavioral responses in freely moving mice. The central amygdala (CeA) was chosen for in vivo magnetothermal deep brain stimulation because it plays a crucial role in physiological and behavioral responses to fearful stimuli [49]. And CeA has been widely used to evaluate the efficiency of various neuromodulation techniques, such as optogenetics [50]. Figure 4a schematically illustrates the process of using anti‐His‐FVIO‐based magnetothermal neurostimulation of the CeA and fear behavior analysis of freely moving mice, whereas an adeno‐associated virus was used to express a His‐tagged TRPV1 and red fluorescent mCherry fusion protein in CeA mouse neurons. After viral transfection, the anti‐His‐FVIO suspension was injected into the CeA region. The immunohistochemical results showed that anti‐His‐FVIO‐FL (green) colocalized with TRPV1‐expressing neurons (red) in the CeA, suggesting that the anti‐His‐FVIOs successfully targeted TRPV1 channels (Figure 4b). After three days of recovery and habituation, the mice were transferred to a custom‐made AMF generator coil equipped with a fear behavior video analysis system. An AMF apparatus with a six‐turn coil (diameter, 12 cm) was used here to produce an AMF with amplitudes up to 20 mT at a frequency of 275 kHz (Figure S11), to guarantee sufficient heat generation by the FVIOs to excite the CeA regions in the deep brains of the mice.

Behavioral changes in the mice in response to different doses of the nano‐mediator were investigated using magnetothermal activation of the CeA. As shown in Figure 4c, 0.05 µg of anti‐His‐FVIOs induced freezing behavior in mice in response to AMF application. The percentage of freezing time was greater during the AMF application period than during the AMF OFF period (Figure 4c). However, none of the mice showed fear behaviors when injected with the same dose of SPIOs (Figure 4d). Further studies revealed that the SPIOs could magnetothermally evoke significant freezing responses only at doses greater than 0.80 µg, which was 16‐fold greater than the dose of FVIOs needed to elicit a response. Moreover, no significant differences in freezing behaviors were noted in the control groups with or without FVIO injection, AMF application, and TRPV1 overexpression. The latency to fear response of the mice was also recorded to analyze the temporal resolution of magnetothermal neurostimulation. Notably, FVIO (0.05 µg)‐treated mice exhibited a fear behavioral response with an average latency time of 2.51 s, which was 2.3‐fold faster than 5.77 s of the SPIO (0.80 µg)‐treated group (Figure 4e). Compared with the previously reported latency time of tens of seconds in mouse experiments, FVIO‐mediated stimulation has been demonstrated to be a superior technique for improving the temporal resolution of in vivo magnetothermal neuromodulation.

The magnetothermal activation of neurons in the CeA was examined using immunoanalysis of c‐fos expression, which is widely used as a measure of neuronal activation. The proportion of cells expressing c‐fos in the CeA was 57.78 ± 3.50% after low‐dose (0.05 µg) FVIO stimulation, as shown in Figure 4f,g. However, a significantly lower percentage of c‐fos‐positive cells (37.16 ± 0.77%) was detected in the high‐dose (0.80 µg) SPIO‐stimulated mice. The results suggested that FVIO‐mediated magnetothermal neurostimulation can efficiently activate many more neurons in the CeA than can SPIO‐mediated neurostimulation under the same conditions.

It is worth noting that the FVIOs are unique magnetic Fe_3_O_4_ nanorings, which possess a ferrimagnetic vortex‐domain structure, in which magnetization is circumferential to the nanoring without stray fields. The FVIOs have negligible remanence and coercivity that can significantly reduce strong dipole–dipole interactions and undesired agglomeration. Thus, the magnetic structure of FVIOs enables the formation of stable suspensions, which is beneficial for stereotactic injection into the CeA region. Importantly, the FVIOs have a much higher saturation magnetization and a large hysteresis loop than that of SPIOs. Under AMF, FVIOs undergo a transition from a vortex state to an onion state remarkably improves the heat‐generating performance of FVIOs in comparison with the most used SPIOs. Therefore, the injectable FVIOs with low doses achieved efficient magnetothermal neuromodulation in vivo.

**FIGURE 5:**
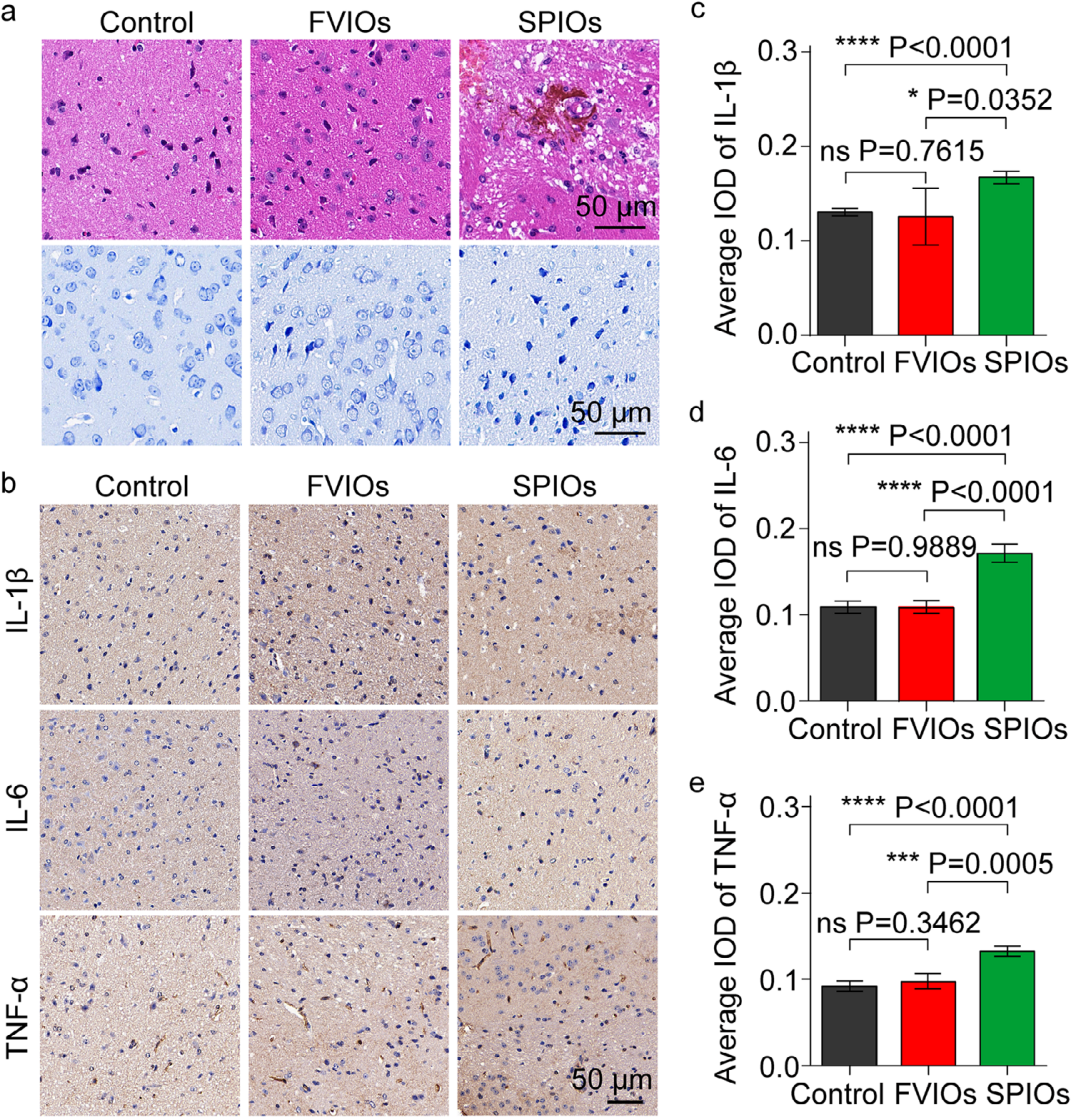
Biosafety evaluation of magnetothermal neurostimulation in vivo. (a) H&E staining (top) and Nissl staining (bottom) of the CeA regions of mice in the control, FVIOs (0.05 µg), and SPIOs (0.80 µg) groups after exposure to the same AMF conditions. Scale bars, 50 µm. (b) Representative immunohistochemical staining pictures of the CeA regions of mice in various groups. The scale bar was 50 µm. Average IOD values in various groups obtained by processing immunohistochemical staining pictures for showing expression levels of the inflammatory factors (c) IL‐1β, (d) IL‐6, and (e) TNF‐α in the CeA regions of mice. Values are mean ± s.e.m. (*n* = 3); one‐way ANOVA (*p < 0.05, **p < 0.01, ***p < 0.001, ****p < 0.0001; ns, not significant).

We tried to establish an optimum FVIO‐mediated stimulation method that satisfies the neuromodulation requirement while resulting in the least tissue damage on the brain region [45]. Therefore, we further evaluated the biosafety of FVIO‐mediated magnetothermal neurostimulation in mice, which is vital for brain safety [51, 52]. Representative hematoxylin and eosin (H&E)‐stained sections from FVIO (0.05 µg)‐treated mice exhibited no histopathological alterations in the CeA region (Figure 5a). In contrast, SPIO treatment at the minimum dose of 0.80 µg caused significant histopathological changes in the CeA regions of the mice, including vacuolar degeneration and nuclear chromatin condensation and fragmentation. After magnetothermal treatment, the SPIOs induced neuronal loss in the CeA regions of the mice, as shown by Nissl staining. More shrunken neurons with pyknotic nuclei were found in the SPIO‐treated mice than in the FVIO‐treated mice (Figure 5a). We further assessed inflammation in the CeA regions of the mice after magnetothemal neurostimulation using immunohistochemical (IHC) analysis (Figure 5b). The expression levels of proinflammatory cytokines (interleukin‐6 (IL‐6), TNF‐α, and IL‐1β) in FVIO‐treated mice were significantly lower than those in SPIO‐treated mice (Figures 5c–e). This was ascribed to the high dose of SPIOs (0.80 µg per CeA region) needed, which caused unfavorable heat damage in the CeA region. These results imply that FVIO‐mediated magnetothermal neurostimulation is much safer than SPIO‐based treatment.

**FIGURE 6:**
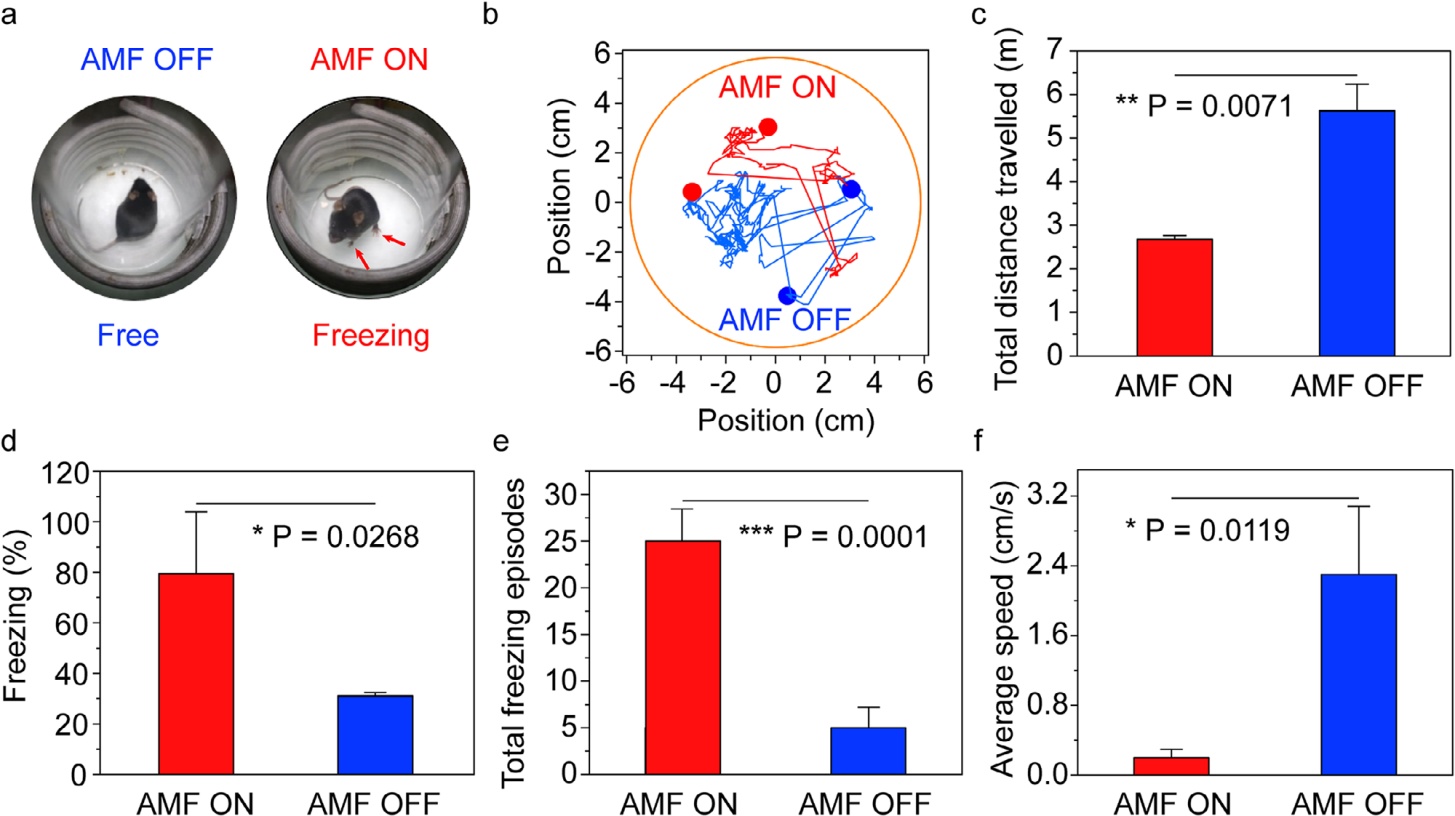
FVIO‐mediated magnetothermal neuromodulation of mouse fear behaviors. (a) A photograph of TRPV1‐expressing mouse inside the AMF coil for anti‐His‐FVIO‐based magnetothermal neurostimulation. Behaviors were observed during AMF ON and OFF conditions (20 mT, 275 kHz, 30 s). (b) Example of tracked positions, (c) total distance traveled, (d) percentage of freezing time, (e) total number of freezing episodes, and (f) average speed among the FVIO‐treated mice in the AMF coil before (AMF OFF) and during (AMF ON) AMF exposure. The data are presented as the mean ± s.e.m. (*n* = 6). The experiments were repeated three times independently.

The fear behaviors induced by magnetothermal treatment in freely moving mice administered the optimal dose of FVIOs were systematically investigated using in situ video recording and further analysis according to standard protocols. Upon AMF exposure, the FVIO‐stimulated mice showed freezing of gait, with locomotion inhibited in place and all four paws locked, although the mice were free to move their heads (Figure 6a). These behaviors reflected the innate anxiety and fear responses of the mice. The tracked positions and total distances traveled are displayed in Figure 6b,c. The mice displayed active locomotion within the arena (coil) before stimulation. During stimulation, the mice preferentially remained frozen, showing an apparent decrease in ambulatory activities (Movie S3). The percentage of freezing time in the FVIO treatment mice after the application of an AMF was 79.58 ± 24.54%, which was 2.56‐fold greater than that among the FVIO‐treated mice without AMF exposure (Figure 6d). Before and during stimulation, the mice also exhibited a significant increase in the total number of freezing episodes (from 5 to 25), which is consistent with the significant decrease in average speed (from 2.3 to 0.2 cm s^−1^), as shown in Figure 6e,f.

### 2.4 Long‐Term Magnetothermal Neurostimulation In Vivo

**FIGURE 7:**
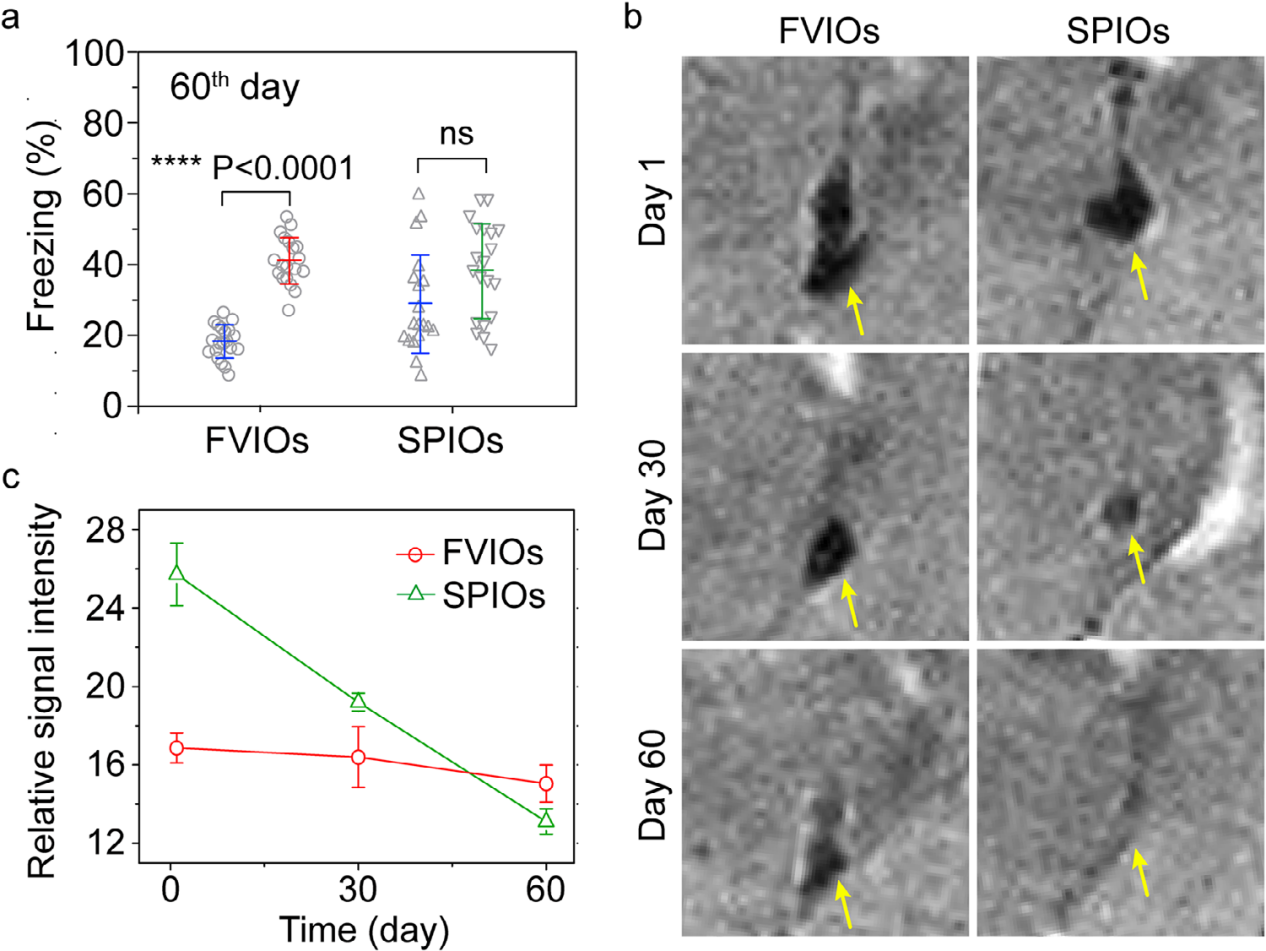
Long‐term magnetothermal neurostimulation mediated by FVIOs. (a) Percentage of freezing time in mice on the 60^th^ day after injection of anti‐His‐FVIOs (or anti‐His‐SPIOs) before and after magnetothermal treatment. The data are presented as the means ± s.e.m. (*n* = 3). (b) UTE MR images of mouse brains were used to evaluate the amount of anti‐His‐FVIOs (or anti‐His‐SPIOs) remaining in the CeA regions on the first, 30^th^, and 60^th^ days. (c) Plots of the relative *T*_2_ signal intensity to noise in the CeA regions in the UTE MR images obtained from FVIO‐ and SPIO‐treated mice.

To evaluate the potential of long‐term and repeated neuromodulation using FVIOs, fear behavior testing in mice was conducted over 60 days. As shown in Figure 7a, the FVIOs retained the ability to magnetothermally elicit freezing behaviors in mice on the 60^th^ day. However, no significant changes in the percentage of freezing time were observed among the SPIO (0.80 µg)‐treated mice, which may be due to SPIO biodegradation in the CeA. To confirm this, 7‐Tesla magnetic resonance imaging (MRI) was employed to evaluate the changes in the concentrations of the magnetic nanoheaters in the CeA regions on the first, 30th, and 60th days using ultrashort echo time (UTE) pulse sequences. Linear correlations of the transverse relaxation rates (1/*T*_2_ values) of the FVIOs and SPIOs were first obtained (Figure S12). Figure 7b presents MR images of mouse brains with which the concentrations of FVIOs and SPIOs that remained in the CeA regions could be determined (marked with yellow arrows) on the first, 30^th^, and 60^th^ days. The relative signal intensity of the FVIOs measured in the CeA remained stable for 60 days (Figure 7c), suggesting that the amount of FVIOs remained unchanged at the injection site. However, the SPIOs signal in the CeA notably decreased on the 60^th^ day, indicating their disappearance. The disappearance of the SPIOs is mainly ascribed to their smaller size in comparison with the FVIOs, which makes their biodegradation and diffusion easier [53]. Previous study demonstrated that SPIOs (<20 nm) were able to diffuse freely through the interstitial space of the brain, being gradually eliminated from the injection site (striatum region) over three weeks [54, 55]. On the other hand, FVIOs with a large size (>20 nm) show slow biodegradation and clearance and remain tightly localized in the closest vicinity of the injection site [56, 57]. Therefore, the FVIOs were highly stable under physiological conditions, giving them great potential for long‐term and repeated neural stimulation and therapy [58].

### 2.5 Transgene‐Free Magnetothermal Neurostimulation and the Induction of Fear Behaviors

**FIGURE 8:**
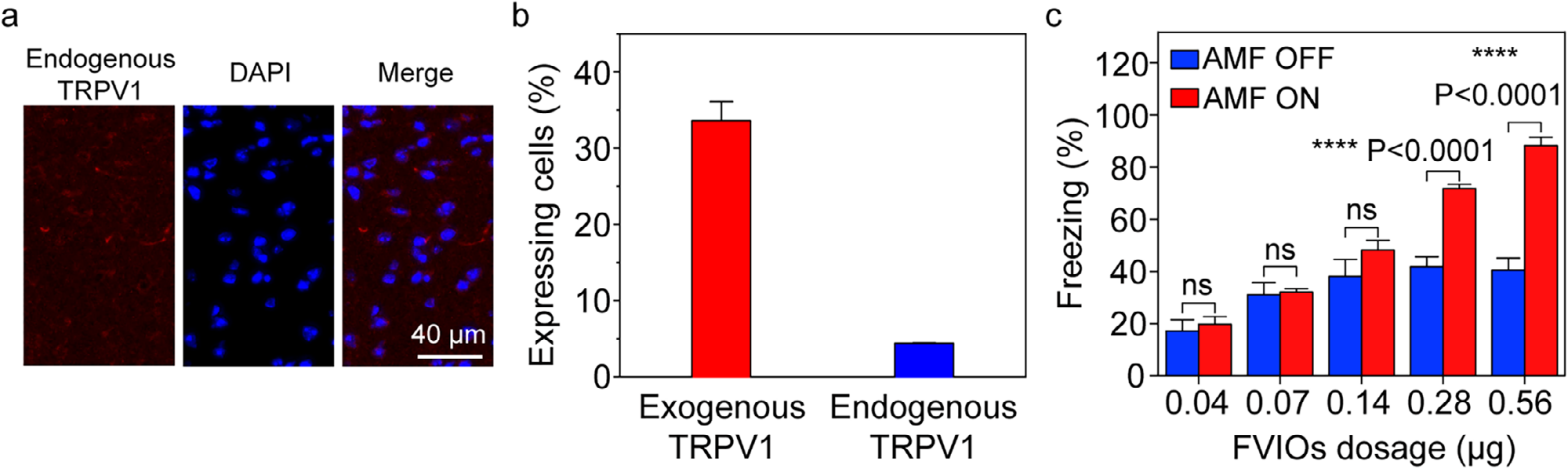
Transgene‐free magnetothermal regulation of fear behaviors in mice using FVIOs. (a) Endogenous TRPV1 expression in the CeA regions of transgene‐free mice visualized by immunofluorescence. (b) Quantification of endogenous and exogenous TRPV1 expression in the CeA regions of transgenic and transgene‐free mice (*n* = 4, means ± s.e.m.). (c) Percentage of freezing time in transgene‐free mice treated with different doses of FVIOs under AMF exposure (*n* = 3 to 5, means ± s.e.m.).

Motivated by the highly efficient activation of neurons in the CeA regions of transgenic TRPV1‐expressing mice by the FVIOs, we further explored the possibility of applying this nanoheater to activate endogenous TRPV1‐expressing neurons in transgene‐free mice. As previously reported, TRPV1 is endogenously expressed on neurons in the CeA of mice [59, 60]. We also confirmed the endogenous expression of TRPV1 (red) on cells in the CeA regions of mice using immunohistochemical methods, as shown in Figure 8a,b. Then, the FVIOs were modified with anti‐TRPV1 antibodies, as confirmed by FTIR spectra (Figure S13), to target endogenous TRPV1 in the CeA regions of transgene‐free mice. Different doses of anti‐TRPV1‐FVIOs were injected into the CeA regions of these mice, and the fear behavioral response induced by FVIO‐mediated magnetothermal stimulation was investigated under AMF exposure (20 mT, 275 kHz). The results verified that the anti‐TRPV1‐FVIOs elicited freezing behaviors in the mice at a minimum dose of 0.28 µg (Figure 8c). This transgene‐free magnetothermal neurostimulation strategy avoids possible side effects arising from the overexpression of exogenous TRPV1 and gene delivery with viral vectors, which further improves the biosafety and expands the scope of magnetothermal neurostimulation applications [11, 61]. However, the expression of TRPV1 in CeA was relatively low. Using novel exogenous thermal‐responsive ion channels is important to promote the development of magnetothermal neurostimulation. Recently, a rate‐sensitive thermoreceptor TRPA1‐A shows sensitivity to the rate of temperature changes of 0.001–0.005°C. Rapid heating of the TRPA1‐A can lower the response threshold, making it an ideal target ion channel to realize efficient magnetothermal neurostimulation in mammals.

## 3 Conclusion

In this work, we established the FVIO‐mediated magnetothermal neurostimulation technique by systematically investigating the effect of FVIOs dose on stimulus‐response time and the in vitro and in vivo biosafety, as well as exploring the effectiveness of using this system for long‐term, repeated stimulation and magnetothermal regulation in the deep brains of transgene‐free mice. Benefiting from the superior heat‐generating performance of the FVIOs, anti‐His antibody‐coated FVIOs triggered Ca^2+^ transients in both transfected 293T cells and cortical neurons at a minimum Fe concentration of 51 µg mL^−1^, which was 20.27‐fold lower than the Fe concentration in the SPIOs. In vivo magnetothermal stimulation of the CeA in freely moving mice demonstrated that the mice treated with FVIOs at the optimal dose of 0.05 µg had a short latency to fear behavior response of approximately 2.51 s, which was 2.3 times faster than that of the SPIO‐treated mice under the same AMF conditions. Furthermore, FVIO‐mediated magnetothermal stimulation was safer and allowed long‐term control of the freezing behaviors of mice for more than 60 days. Even in nongenetically modified mice, 0.28 µg of FVIOs was able to activate the endogenous TRPV1 in the CeA and elicit fear behaviors. Overall, we believe that the FVIO‐mediated efficient and safe neuromodulation technique established in this study has potential for future neuroscience and therapeutic applications.

## 4 Materials and Methods

### 4.1 Materials

The reagents 3‐(3,4‐dihydroxyphenyl) propanoic acid (DHCA), fluorescein isothiocyanate (FL), *N*‐ethyl‐*N′*‐(3‐dimethylaminopropyl) carbodiimide (EDC), and *N*‐hydroxysuccinimide (NHS) were obtained from Sigma‐Aldrich (Shanghai, China). Capsaicin and capsazepine were purchased from Aladdin (Shanghai, China). Fluo 4‐AM was purchased from Invitrogen (Shanghai, China).

### 4.2 Synthesis and Characterization of FVIOs

The FVIOs were synthesized according to our previously reported method. Briefly, the hematite *α*‐Fe_2_O_3_ nanorings were prepared through hydrothermal treatment of FeCl_3_·6H_2_O and NH_4_H_2_PO_4_ solutions at 220°C for 48 h. The *α*‐Fe_2_O_3_ nanorings were transformed to FVIOs (Fe_3_O_4_ nanorings) in a 20% H_2_/80% Ar atmosphere at 400°C for 3 h. The FVIOs were dispersed into 30 mL of oleic acid, and the mixture was heated and maintained at 260°C for 1 h under an Ar flow. After cooling to room temperature, the oleic acid‐coated FVIOs were obtained after washing and centrifuging with an ethanol/hexane solution.

*Phase Transfer*: The aqueous FVIOs suspension was prepared by coating DHCA on the surface of nanorings via a ligand exchange of oleic acid. Oleic acid‐coated FVIOs (10 mg) and DHCA (500 mg) were dispersed in a THF solution in a three‐neck flask, which was stirred at 65°C for 5 h under Ar flow. The DHCA‐modified FVIOs (DHCA‐FVIOs) were washed and centrifuged with deionized water and stored in PBS (0.1 M, pH 7.4) for further experiments.

*Antibody binding*: The anti‐His antibody was covalently linked to FVIOs via EDC/NHS reaction. DHCA‐FVIOs (1 mg) were reacted with EDC (10 mM) and NHS (10 mM) for 15 min in MES buffer (0.1 M, pH 6.0). Then, the solution was adjusted to pH 7.4, and 5 µL of mouse monoclonal anti‐His antibody was added and shaken for 2 h. The final products were washed with PBS to remove unbound antibodies and dispersed in PBS before in vitro and in vivo testing.

*Characterization*: The crystal structure of magnetic nanoheaters was characterized by XRD patterns using a powder diffractometer (Bruker, D8 Advance, Germany). The morphology of nanoheaters was observed by scanning electron microscopy (SEM, Hitachi SU8010, Japan). The anti‐His antibody‐coated nanoheaters were stained with phosphotungstic acid and characterized by transmission electron microscopy (TEM, HT‐7700, Japan). The hydrodynamic diameters of nanoheaters were examined by dynamic light scattering (Malvern, England). The Fe concentration of nanoheater suspensions was detected using inductively coupled plasma mass spectrometry (Agilent 7900, America). The magnetization curves were determined using a VSM (BKT‐4600, China).

### 4.3 Molecular Cloning and Virus Packing

The CMV promoter drives the expression of TRPV1 and mCherry, which were separated by a 2A sequence followed by a stop sequence. TRPV1 DNA fragments inserted with a 6 × His tag were synthesized and cloned into plasmids. The plasmid was packed into the Lenti virus according to established protocols. Before use, all the viral vectors were diluted to a titer of 10^12^ transducing units per milliliter and used for neuronal cell transduction.

### 4.4 Cell Culture and Transfection

HEK293T cell culture: Before in vitro experiments, the cells were authenticated and checked for mycoplasma contamination. Human embryonic kidney (HEK293T) cells were plated sparsely on culture dishes and cultured in Dulbecco's modified Eagle's medium (DMEM) supplemented with 10% fetal bovine serum (FBS) at 37°C under 5% CO_2_. For calcium imaging, the cells were carefully transfected with a certain number of plasmids encoding TRPV1 using 1 µL of a standard transfection reagent (GenEscort III) with 2 µg of total DNA. Magnetothermal stimulation was performed one day after transfection of the genetically encoded 293T cells.

*Cortical neural cell culture*: The whole brain was transferred to PBS containing glucose (33 mM), penicillin‐streptomycin (1%, v/v), and washed. The cortical tissues were dissected in a trypsin solution (0.25%, 37°C, 30 min), which was quenched using horse serum (10%, Fisher Scientific) in neurobasal medium. The dissociated tissues were triturated and filtered. The obtained cells were plated onto poly‐L‐lysine‐coated culture dishes. Unattached cells were removed after 2 h of incubation. The attached cells were cultured in neurobasal medium containing B‐27, and half of the medium was replaced every 2 days. Five‐day‐old cortical neural cells were transfected by adding 2 µL of Lenti‐TRPV1‐p2A‐mCherry (1 × 10^12^ transducing units mL^−1^). After a 5‐day induction period, magnetothermal stimulation was performed for calcium imaging of the TRPV1‐expressing cortical neural cells.

### 4.5 Cytotoxicity Assay

Cell viability was measured by a CCK‐8 assay in 96‐well plates. FVIOs at various concentrations (0–1000 µg mL^−1^) were added into each well that was seeded with HEK293T cells 24 h prior. Then, the FVIOs were removed from the culture media. Then, 100 µL of CCK‐8 (mg mL^−1^ in DMEM) was added into each well and incubated for 2 h. The absorbance of the supernatant was assayed using a 96‐well plate reader at 450 nm.

### 4.6 In Vitro Magnetothermal Stimulation and Calcium Imaging

Calcium imaging in vitro and fluorescence change quantification were performed as follows. The TRPV1^His^‐expressing HEK293T cells were washed three times in PBS and incubated with Fluo‐4 (2 µM) for 30 min at 37°C. Then, the cells were rewashed with PBS. Next, the anti‐His‐FVIOs solution (1 mg mL^−1^) was added to the culture medium of TRPV1‐expressing cells on a dish for 15 min incubation. The unbound FVIOs in cell culture medium were washed and removed with calcium imaging buffer (105 mM NaCl, 3 mM KCl, 2.5 mM CaCl_2_, 0.6 mM MgCl_2_, 10 mM HEPES, 1.2 mM NaHCO_3_, 100 mM mannitol, and 10 mM glucose) and then imaged in calcium imaging buffer before and during AMF treatment. A magneTherm system with a live‐cell coil was employed to provide an AMF at 290 kHz and 20 mT. Calcium fluorescence imaging was performed on a confocal laser microscope (Nikon) system at 20× magnification. The fluorescence intensities (*F_t_*) during AMF exposure were normalized to the average baseline fluorescence (*F*_0_) to calculate the relative fold change *F_t_*/*F*_0_. The *F*_0_ for each examined cell was obtained for the first 20 s before AMF treatment. Responsive cells and the standard deviations of signals were determined during the stimulation period. The cultured TRPV1^His^‐expressing cortical neurons that bound with FVIOs were also magneto‐thermally stimulated for calcium imaging experiment as the protocol.

### 4.7 Mice and Viral Expression in CeA Regions

Adult male C57BL/6 mice (6–8 weeks, 20–25 g, Vital River Inc., Beijing, China) were used in this study. Mice were housed 6 per cage in the animal facility and were free to food and water. Surgeries, including virus and nanoheater injection, were conducted using a standard stereotaxic apparatus under aseptic conditions. The mice were anesthetized through an intraperitoneal injection of pentobarbital sodium (40 mg kg^−1^). Before in vivo magnetothermal neurostimulation, AAV5 virus encoding TRPV1^His^‐mCherry was packaged and injected into CeA region for getting TRPV1^His^‐expressing mice. The stereotaxic coordinate for the CeA was as follows: – 1.25 AP, – 2.75 ML, – 4.30 DV. A small hole was drilled in the skull at the coordinate of CeA and a 32‐gauge needle was lowered into the hole. 300 nL of AAV5 virus was injected into the CeA region at 0.1 µL min^−1^ using a microsyringe pump. After being lifted by 0.1 mm, the syringe was remained for 10 min within the brain before slow withdrawal. At least 4 weeks postviral expression, the anti‐His‐FVIOs in PBS (2 mg mL^−1^) were injected into the same CeA region. The skin tissue was closed with sutures. TRPV1‐expressing and FVIOs‐loaded mice were prehoused in a closed box with a diameter of 12 cm under a reverse 12‐h light/dark cycle for 3 days as a habituation stage. Animal husbandry and all experimental manipulation of the animals were performed with the approval (Approved No. 2020311) of the Animal Care and Use Committee of the Northwest University.

### 4.8 In Vivo Magnetothermal Stimulation and Fear Behavior Analysis

The AMF exposure was set to 20 mT and 290 kHz for the magnetothermal genetic stimulation experiment. Cameras automatically recorded animal behaviors, and their movement trajectories were analyzed using Any‐Maze software. Total freezing time during each trial was used as an index of fear behaviors and converted to a percentage for analysis. The same contexts (odors, wall, color, flooring, and sound‐attenuating cabinet) were used to minimize contextual freezing. The percentage of freezing time spent during each FVIOs‐treated trial was compared across groups (including SPIOs‐treated, without AMF exposure).

### 4.9 Histology

Histology was used to detect the expression of TRPV1 and c‐fos and the successful targeting of anti‐His‐FVIOs to the TRPV1‐expressing neurons. After AMF treatment, the mice were anesthetized and perfused transcardially with PBS and 4% paraformaldehyde (PFA) within 1.5 h. The isolated brains were fixed in 10% PFA at 4°C overnight and equilibrated for cryoprotection in 30% sucrose. Coronal brain slices (40 µm thick) were cut on a vibratome. The mouse anti‐His tag monoclonal antibody and the mouse monoclonal anti‐TRPV1 antibody were used in histology. The obtained slices were stained and photographed using a laser‐scanning confocal microscope. Confocal images were analyzed using ImageJ software.

## Conflicts of Interest

The authors declare no conflicts of interest.

## Supporting information




**Supporting File 1**: exp270096‐sup‐0001‐SuppMat.docx

**Supporting File 2**: exp270096‐sup‐0001‐SuppMat.docx

**Supporting File 3**: exp270096‐sup‐0001‐SuppMat.docx

**Supporting File 4**: exp270096‐sup‐0001‐SuppMat.docx

## Data Availability

All data related to this work are present in the article and the Supporting Information. Any other data associated with this study are available from the corresponding authors upon request.
