# Supplementary material for: Ferrimagnetic Vortex Nanorings Facilitate Efficient and Safe Deep‐Brain Magnetothermal Stimulation in Freely Moving Mice
Source: Exploration (Beijing). 2025 Dec 4;5(6):20240118. doi: 10.1002/EXP.20240118 (PMC12752603; doi:10.1002/EXP.20240118)
Supplement: Supplementary file 1 — Supporting File 1: exp270096‐sup‐0001‐SuppMat.docx [file EXP2-5-20240118-s002.docx]

**Supplementary Materials for**

**Ferrimagnetic Vortex Nanorings Facilitate Efficient and Safe Deep-Brain Magnetothermal Stimulation in Freely Moving Mice**

Galong Li^1, 3, #^, Xin Qiao^2, #^, Yu Zhao^4^, Dongyan Li^1^, Guigen Zhang^4^, Xiaoli Liu^1^, Fulin Chen^1^, Huaning Wang^5^, Hongbing Lu^3^, Jin Zhou^2,^ *, Changyong Wang^2,^ *, Haiming Fan^1,^ *

^1^ College of Chemistry and Materials Science, Key Laboratory of Synthetic and Natural Functional Molecule of Ministry of Education, Northwest University, Xi'an, China

^2^ Beijing Institute of Basic Medical Sciences, 27 Taiping Rd, Beijing, 100850, PR China

^3^ School of Biomedical Engineering, Shaanxi Provincial Key Laboratory of Bioelectromagnetic Detection and Intelligent Perception, Air Force Medical University, Xi'an, China

^4^ Department of Biomedical Engineering, University of Kentucky, Lexington, KY, 40506 USA

^5^ Department of psychiatry, Xijing Hospital, Air Force Medical University, Xi'an, China

* Corresponding author.

E-mail address: fanhm@nwu.edu.cn (H. Fan)

wcy2000_zm@163.com (C. Wang)

sisun820819@163.com (J. Zhou)

^#^ Galong Li and Xin Qiao contributed equally to this work.


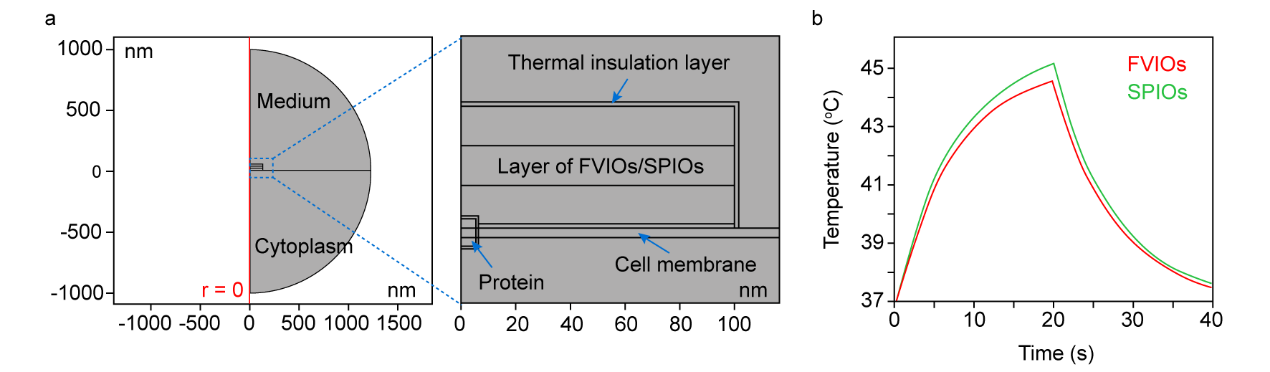


Figure S1. The finite-element modeling was used to predict local temperature changes near a TRPV1 channel during the magnetothermal activation. It was found that FVIOs (8.70 × 10^-6^ ng per TRPV1) and SPIOs (3.36 × 10^-5^ ng per TRPV1) were needed to generate a local temperature increase from 37 to 43 ^o^C. Thus, FVIOs and SPIOs can achieve similar heating activation of a single TRPV1 channel under the same AMF conditions. (a) 2D axisymmetric model for magnetothermal activation of a single TRPV1 channel on cell membrane. The thermal insulation layer behaves like interfacial thermal resistance with a 0.1 m^2^ **·** K/W resistivity. The temperature of the outer boundary of the model is 37 ^o^C. (b) Simulated temperature increase profile for magnetothermal activation of a single TRPV1 channel using FVIOs and SPIOs.


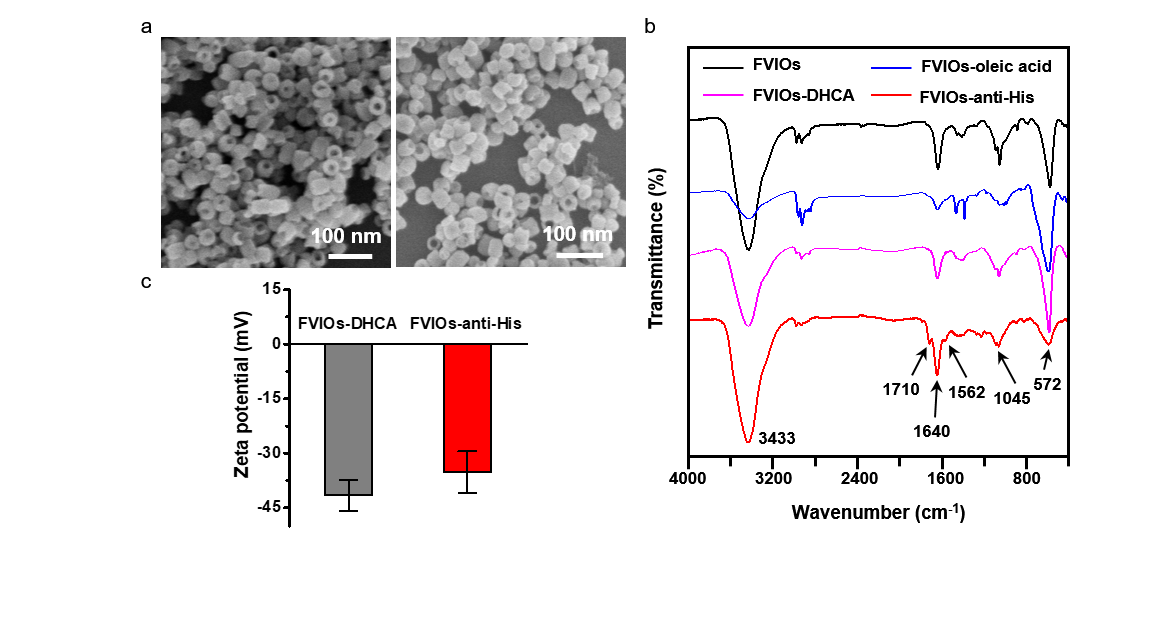


Figure S2. (a) Scanning electron micrograph of FVIOs (left) and FVIOs-DHCA (right). (b) FTIR spectra of FVIOs, FVIOs-oleic acid, FVIOs-DHCA, and FVIOs-anti-His antibody. The appearance of the peaks at 3433 cm^-1^, 1640 cm^-1^, 1045 cm^-1^, and 572 cm^-1^ for FVIOs-anti-His antibody, which correspond to the –N–H– stretching vibration of amides, C=O stretching vibrations, C–O flexural vibrations, Fe–O flexural vibrations, respectively. New peaks of 1710 cm^-1^ and 1562 cm^-1^ can be ascribed to the C=O (amide I bond) and C–N and N–H (amide II bands), respectively. This result indicates the formation of amide covalent binding, which occurs from the reaction between the –COOH groups of FVIOs and –NH_2_ groups of the anti-His antibody with the use of EDC/NHS reaction. (c) Zeta potentials of FVIOs-DHCA and FVIOs-anti-His antibody are −41 ± 4 mV and −35 ± 5 mV, respectively, which confirms the conjugation of anti-His antibody on FVIOs.


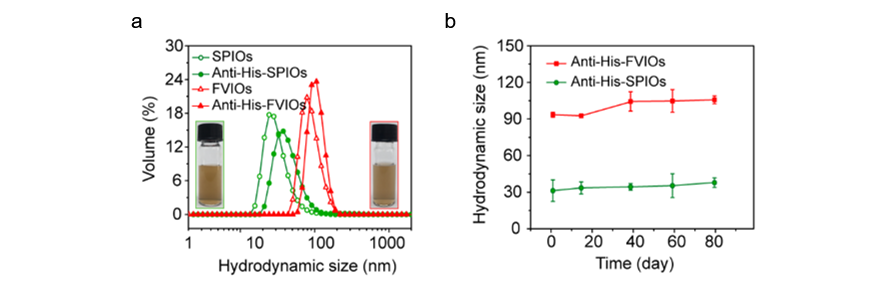


Figure S3. (a) Dynamic light scattering spectra of the FVIOs, anti-His-FVIOs, SPIOs, and anti-His-SPIOs. (b) Hydrodynamic size changes of FVIOs and SPIOs suspensions during the 1^st^ to 80^th^ day.


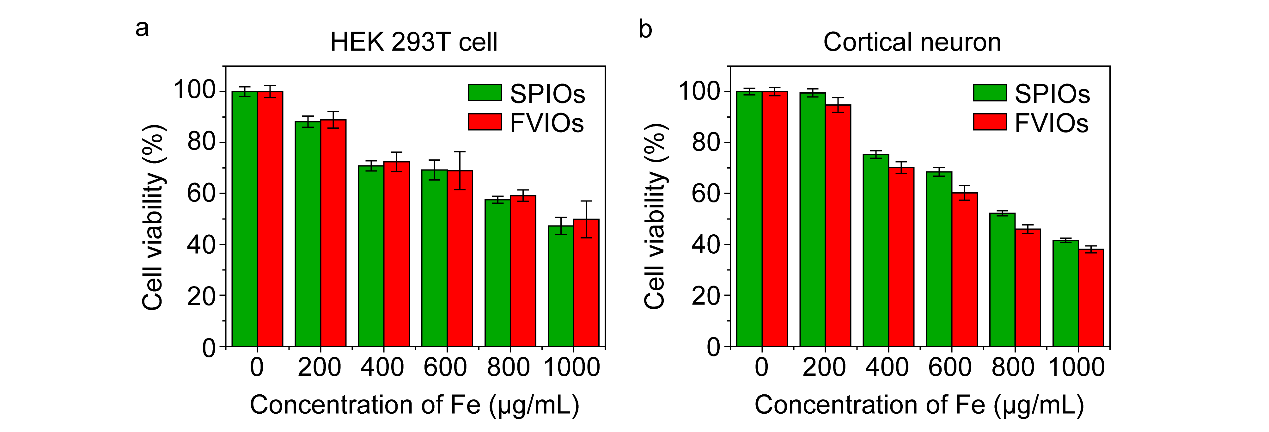


Figure S4. (a,b) Cell viability as a function of Fe concentration (FVIOs, SPIOs) from 0 to 1000 ug mL^-1^ within cells growth media.


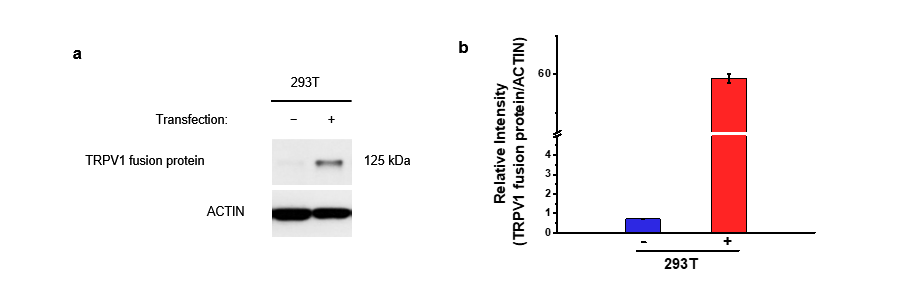


Figure S5. (a) Western blot analysis of TRPV1 fusion protein expressed on HEK293T cells, and (b) relative protein expression of TRPV1 to ACTIN.


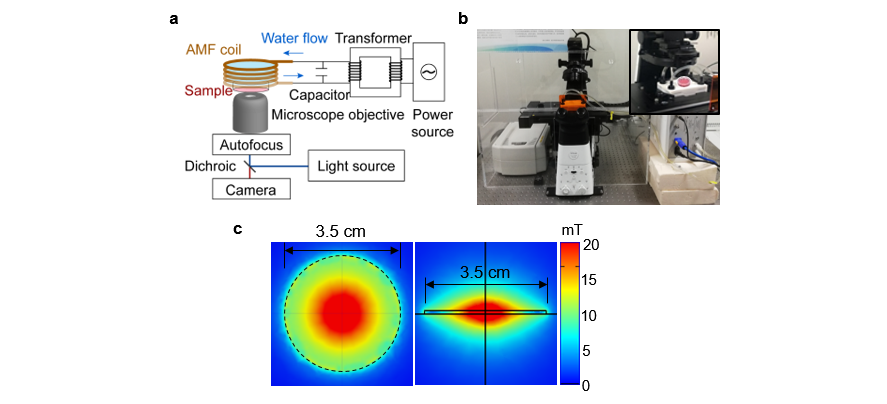


Figure S6. (a) Illustration of the in vitro experimental setup combining the alternating magnetic field (AMF) application with confocal laser scanning microscopy (CLSM). The AMF is equipped with continuously water-cooled coil made of copper pipe. The AMF coil and capacitor form an electrical resonator that is driven by an alternating power source. Cells grown on confocal dish are placed directly underneath the coil. Any focus drifts are compensated by a fast laser autofocus. (b) A photograph of the in vitro experimental setup combining AMF with confocal microscopy. (c) The color map represents the magnetic flux distribution calculated by a finite element model for the AMF coil.


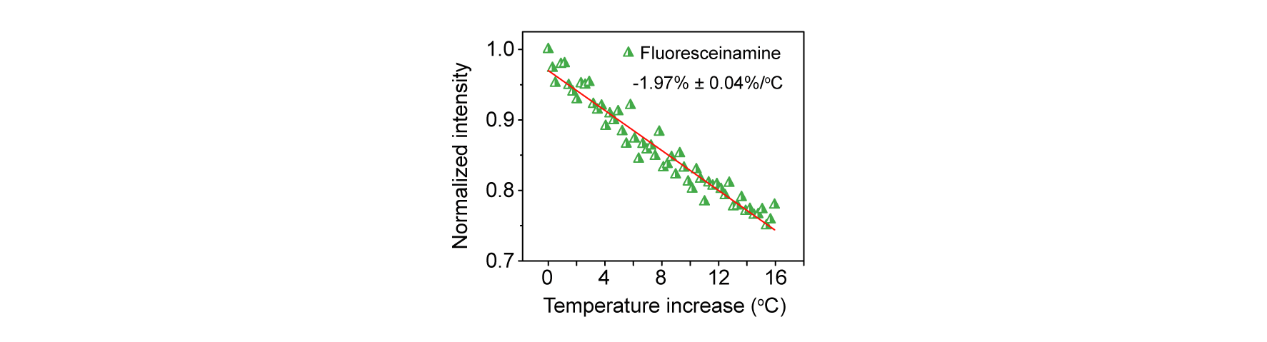


Figure S7. A linear decay was found in the percentile change of fluorescence intensity with temperature increase, showing 1.97 ± 0.04 %/^o^C for fluoresceinamine (FL).


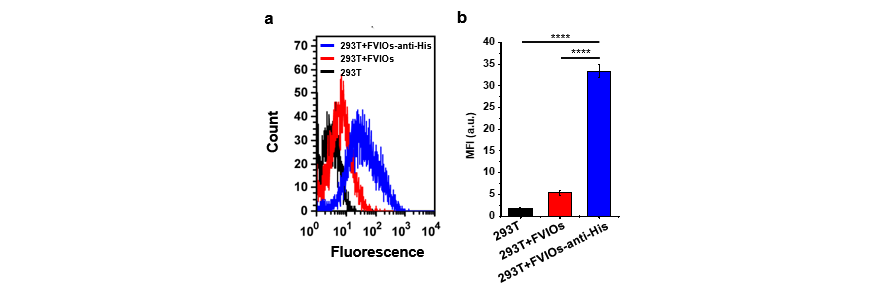


Figure S8. (a,b) Flow cytometric assay for the binding of FVIOs-anti-His-FL with TRPV1-expressing HEK293T cells.


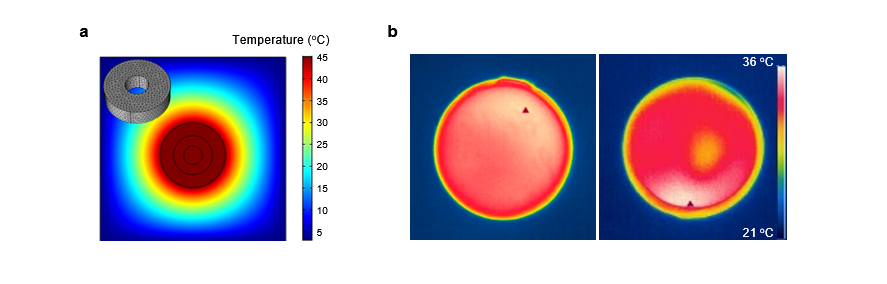


Figure S9. (a) The color map represents the temperature distribution on the surface of an FVIO as calculated by Comsol Multiphysics about the heat transfer studies. (b) Infrared thermal images of the cell-cultured confocal dish before and after AMF exposure for in vitro experiments.


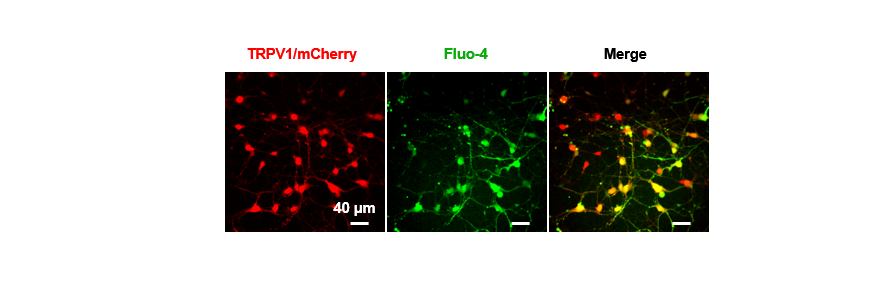


Figure S10. Confocal images of cortical neuron cells expressing TRPV1 and loaded with Fluo-4 for further FVIO-mediated magnetothermal activation.


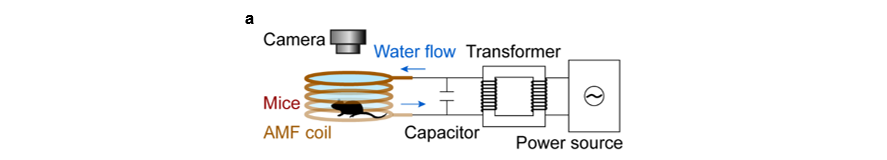


Figure S11. (a) The experimental setup for in vivo magnetothermal neurostimulation of mice. The AMF coil around the arena generated the AMF. An overhead camera was used to record the mouse’s behavior in the arena.


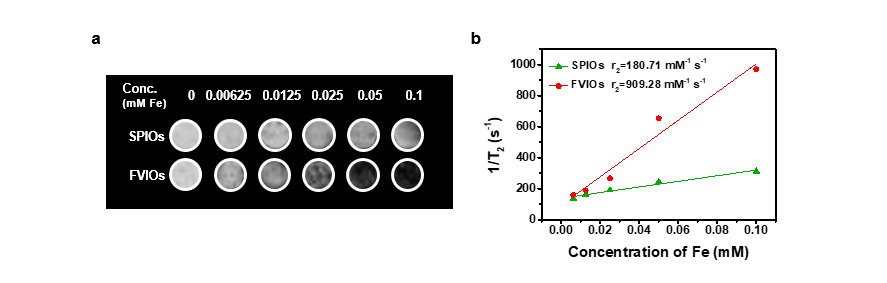


Figure S12. (a) T_2_ phantom images of FVIOs and SPIOs. (b) Plots of 1/T_2_ over Fe concentration of the FVIOs and SPIOs. The slope indicates the specific relaxivity (r_2_).


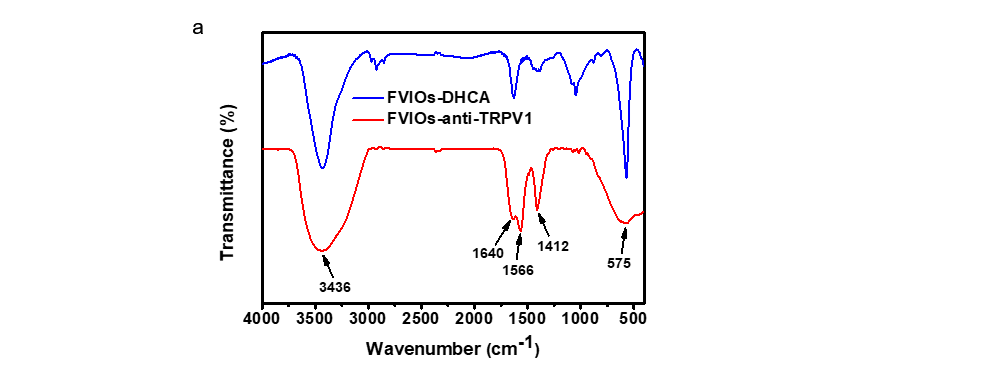


Figure S13. (a) FTIR spectra of FVIOs-DHCA and FVIOs-anti-TRPV1 antibody. The appearance of the peaks at 3436 cm^-1^ and 575 cm^-1^ for FVIOs-anti-TRPV1 antibody, which correspond to the –N–H– stretching vibration of amides, and Fe–O flexural vibrations, respectively. New peaks of 1640 cm^-1^ and 1566 cm^-1^ can be ascribed to the C=O (amide I bond) and C–N and N–H (amide II bands), respectively. This result indicates the formation of amide covalent binding, which occurs from the reaction between the –COOH groups of FVIOs and –NH_2_ groups of the anti-TRPV1 antibody.
